# Methyl 2-[2-(benzyl­oxycarbon­ylamino)­propan-2-yl]-5-hy­droxy-1-methyl-6-oxo-1,6-dihydro­pyrimidine-4-carboxyl­ate

**DOI:** 10.1107/S160053681204233X

**Published:** 2012-10-20

**Authors:** Zhenhua Shang, Xiao Tao, Jing Ha, Fuda Yu

**Affiliations:** a College of Chemical and Pharmaceutical Engineering, Hebei University of Science and Technology, Hebei Research Center of Pharmaceutical and Chemical Engineering, State Key Laboratory Breeding Base-Hebei Province, Key Laboratory of Molecular Chemistry for Drugs, Shijiazhuang 050018, People’s Republic of China; b College of Chemical and Pharmaceutical Engineering, Hebei University of Science and Technology, Shijiazhuang 050018, People’s Republic of China; c Zhongqi Pharmacy (Shijiazhang), Shijiazhuang Pharmaceutical Group Co., Ltd (CSPC), Shijiazhuang 050051, People’s Republic of China; d Department of Neurosurgery, Shijiazhuang Center Hospital, Shijiazhuang 050011, People’s Republic of China

## Abstract

The title pyrimidine derivative, C_18_H_21_N_3_O_6_, was obtained by the reaction of methyl 2-[2-(benzyl­oxycarbon­yl)amino­propan-2-yl]-5-hy­droxy-6-oxo-1,6-dihydro­pyrimidine-4-carboxyl­ate with dimethyl sulfate in dimethyl sulfoxide. The mol­ecule has a V-shaped structure, the phenyl and the pyrimidine rings making a dihedral angle of 43.1 (1)°. The methyl group substituting the pyrimidine ring deviates slightly from the ring mean-plane [C—N—C—C torsion angle = 5.49 (15)°], and the methyl ester substituent has a conformation suitable for the formation of an intra­molecular O—H⋯O hydrogen bond with the hydroxyl functionality. In the crystal, molecules are linked into chains along the *b* axis by N—H⋯O hydrogen bonds.

## Related literature

For the anti­retroviral drug raltegravir [systematic name: *N*-(2-(4-(4-fluoro­benzyl­carbamo­yl)-5-hy­droxy-1-methyl-6-oxo-1,6-dihydro­pyrimidin-2-yl)propan-2-yl)], see: Steigbigel *et al.* (2008 ▶). For the synthesis of raltegravir, see: Belyk *et al.* (2006 ▶); For related structures, see: Fun *et al.* (2011 ▶); Shang, Ha, Yu & Zhao (2011 ▶); Shang, Qi, Tao & Zhang (2011 ▶).
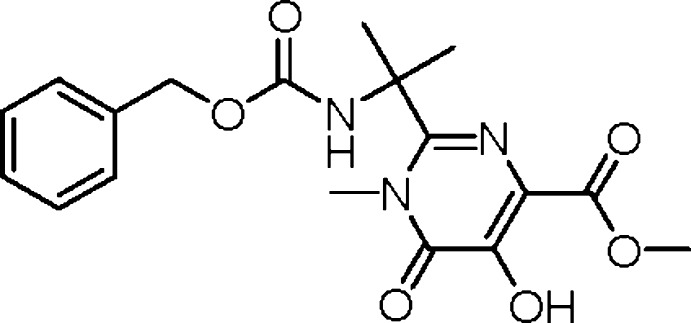


## Experimental

### 

#### Crystal data

C_18_H_21_N_3_O_6_
*M* *_r_* = 375.38
Monoclinic, 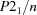

*a* = 10.540 (2) Å
*b* = 12.927 (3) Å
*c* = 13.751 (3) Å
β = 109.74 (3)°
*V* = 1763.5 (6) Å^3^
*Z* = 4
Mo *K*α radiation
μ = 0.11 mm^−1^
*T* = 113 K
0.20 × 0.16 × 0.12 mm

#### Data collection

Rigaku Saturn diffractometer
Absorption correction: multi-scan (*CrystalClear*; Rigaku/MSC, 2005 ▶) *T*_min_ = 0.979, *T*_max_ = 0.987
12669 measured reflections
4184 independent reflections
2889 reflections with *I* > 2σ(*I*)
*R*_int_ = 0.032

#### Refinement

*R*[*F* ^2^ > 2σ(*F* ^2^)] = 0.035
*wR*(*F* ^2^) = 0.093
*S* = 1.00
4184 reflections
255 parameters
H atoms treated by a mixture of independent and constrained refinement
Δρ_max_ = 0.28 e Å^−3^
Δρ_min_ = −0.20 e Å^−3^

### 

Data collection: *CrystalClear* (Rigaku/MSC, 2005 ▶); cell refinement: *CrystalClear*; data reduction: *CrystalClear*; program(s) used to solve structure: *SHELXS97* (Sheldrick, 2008 ▶); program(s) used to refine structure: *SHELXL97* (Sheldrick, 2008 ▶); molecular graphics: *SHELXTL* (Sheldrick, 2008 ▶); software used to prepare material for publication: *CrystalStructure* (Rigaku/MSC, 2005 ▶).

## Supplementary Material

Crystal structure: contains datablock(s) global, I. DOI: 10.1107/S160053681204233X/bh2453sup1.cif

Structure factors: contains datablock(s) I. DOI: 10.1107/S160053681204233X/bh2453Isup2.hkl

Supplementary material file. DOI: 10.1107/S160053681204233X/bh2453Isup3.cml

Additional supplementary materials:  crystallographic information; 3D view; checkCIF report

## Figures and Tables

**Table 1:** Hydrogen-bond geometry (Å, °)

*D*—H⋯*A*	*D*—H	H⋯*A*	*D*⋯*A*	*D*—H⋯*A*
O2—H2⋯O3	0.876 (13)	1.782 (14)	2.5879 (12)	151.9 (14)
N3—H3⋯O1^i^	0.875 (15)	2.118 (15)	2.9854 (16)	170.9 (12)

Symmetry code: (i) 
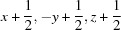
.

**Fig. 1.**
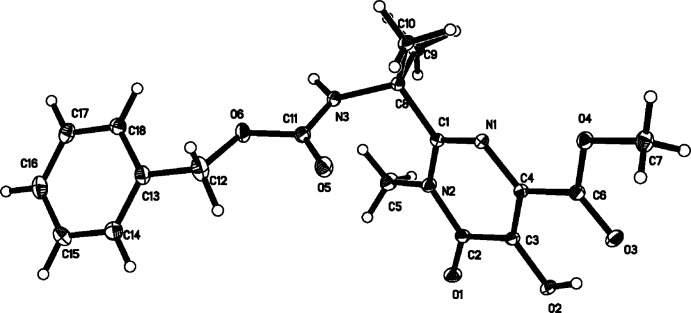
The molecular structure of the title compound, drawn with 30% probability ellipsoids.

## supplementary crystallographic information

### Comment

Pyrimidine derivatives are important chemotherapeutic agents, and Raltegravir (MK-0518, brand name Isentress), an antiretroviral drug produced by Merck & Co, used to treat HIV infection (Steigbigel *et al.*, 2008), is one of the representatives. When methyl 2-[2-(benzyloxycarbonyl)aminopropan-2-yl]-5-hydroxy-6-oxo-1,6-dihydropyrimidine-4-carboxylate was reacted with dimethyl sulfate and magnesium methoxide as catalyst in dimethyl sulfoxide (Belyk *et al.*, 2006), as we designed, in order to synthesize the title compound as the key intermediate of Raltegravir, two products appeared unfortunately. The products were separated by flash chromatography and the structure of the title compound was confirmed by NMR and X-ray analysis. The X-ray results (Fig. 1) showed that the phenyl and pyrimidine rings are not in a common plane, as found in related compounds (Fun *et al.*, 2011; Shang, Ha, Yu & Zhao, 2011; Shang, Qi, Tao & Zhang, 2011). The dihedral angle between the two aromatic rings is 43.1 (1)°. The carbamate unit (atoms C11, N3, O5 and O6) is planar. The methyl group bonded to N2 is slightly deviated from the pyrimidine mean-plane, with the torsion angle C5—N2—C1—C8 = 5.49 (15)°. The conformation of the carboxylate in the methyl ester group is indicated by torsion angles C7—O4—C6—C4 = 179.58 (8)° and C3—C4—C6—O3 = -1.39 (15)°. The crystal structure is stabilized mainly through intermolecular N—H···O and intramolecular O—H···O hydrogen bonds.

### Experimental

To a slurry of methyl 2-[2-(benzyloxycarbonyl)aminopropan-2-yl]-5-hydroxy-6-oxo-1,6-dihydropyrimidine-4-carboxylate (1.5 g) and magnesium methoxide (2.1 g) in dimethyl sulfoxide (15 ml) at 70 °C, dimethyl sulfate (3.1 g) was added droply. After addition, the mixture was heated at the same temperature for 8 h. The reaction mixture was then added to 40 ml of HCl 2 N, and then to 100 ml of water. A solid phase appeared when the mixture was stirred with ice-water bath. The products were filtered and separated by flash chromatography. The title compound (50 mg) was dissolved in 50 ml of ethanol at room temperature and the solvent was slowly evaporated over 10 days, affording colourless single crystals suitable for X-ray analysis. ^1^H-NMR (500 MHz, CDCl_3_): 1.75 (s, 6H), 4.05 (s, 3H), 4.07 (s, 3H), 5.12 (s, 2H), 6.58 (bs, 1H), 7.26–7.38 (m, 5H, *J*=18.5 Hz), 10.46 (s, 1H). IR (KBr) 1150, 1240, 1282, 1492, 1454, 1694, 1738, 2975, 3233, 3398 cm^-1^.

### Refinement

All H atoms attached to C atoms were placed geometrically and treated as riding atoms, with C—H = 0.95 (aromatic), 0.98 (methyl CH_3_ group), or 0.99 Å (methlyene group), with *U*_iso_(H)=1.2*U*_eq_(carrier C), or *U*_iso_(H)=1.5*U*_eq_(carrier C). The positions for atoms H2 and H3, bonded to O2 and N3, were refined freely with bond lengths converging to N3—H3 = 0.875 (15) Å and O2—H2 = 0.876 (13) Å.

### Crystal data

**Table:**

C_18_H_21_N_3_O_6_	*F*(000) = 792
*M**_r_* = 375.38	*D*_x_ = 1.414 Mg m^−^^3^
Monoclinic, *P*2_1_/*n*	Mo *K*α radiation, λ = 0.71073 Å
Hall symbol: -P 2yn	Cell parameters from 5478 reflections
*a* = 10.540 (2) Å	θ = 2.1–27.9°
*b* = 12.927 (3) Å	µ = 0.11 mm^−^^1^
*c* = 13.751 (3) Å	*T* = 113 K
β = 109.74 (3)°	Plate, colourless
*V* = 1763.5 (6) Å^3^	0.20 × 0.16 × 0.12 mm
*Z* = 4	

### Data collection

**Table:**

Rigaku Saturn diffractometer	4184 independent reflections
Radiation source: rotating anode	2889 reflections with *I* > 2σ(*I*)
Confocal monochromator	*R*_int_ = 0.032
Detector resolution: 7.31 pixels mm^-1^	θ_max_ = 27.9°, θ_min_ = 2.1°
ω and φ scans	*h* = −13→13
Absorption correction: multi-scan (*CrystalClear*; Rigaku/MSC, 2005)	*k* = −16→16
*T*_min_ = 0.979, *T*_max_ = 0.987	*l* = −18→10
12669 measured reflections	

### Refinement

**Table:**

Refinement on *F*^2^	Primary atom site location: structure-invariant direct methods
Least-squares matrix: full	Secondary atom site location: difference Fourier map
*R*[*F*^2^ > 2σ(*F*^2^)] = 0.035	Hydrogen site location: inferred from neighbouring sites
*wR*(*F*^2^) = 0.093	H atoms treated by a mixture of independent and constrained refinement
*S* = 1.00	*w* = 1/[σ^2^(*F*_o_^2^) + (0.0545*P*)^2^] where *P* = (*F*_o_^2^ + 2*F*_c_^2^)/3
4184 reflections	(Δ/σ)_max_ = 0.001
255 parameters	Δρ_max_ = 0.28 e Å^−^^3^
0 restraints	Δρ_min_ = −0.20 e Å^−^^3^
0 constraints	

### Fractional atomic coordinates and isotropic or equivalent isotropic displacement parameters (Å^2^)

**Table:**

	*x*	*y*	*z*	*U*_iso_*/*U*_eq_	
N1	0.06467 (9)	0.00568 (6)	0.24005 (7)	0.0175 (2)	
N2	0.12798 (9)	0.17823 (6)	0.22657 (8)	0.0184 (2)	
N3	0.23993 (10)	0.16037 (7)	0.45582 (8)	0.0200 (2)	
O1	0.01828 (8)	0.27591 (6)	0.08460 (7)	0.0245 (2)	
O2	−0.13087 (8)	0.11324 (6)	−0.01134 (6)	0.02320 (19)	
H2	−0.1711 (14)	0.0529 (10)	−0.0243 (12)	0.035*	
O3	−0.18898 (8)	−0.07843 (6)	0.00815 (7)	0.0248 (2)	
O4	−0.07643 (7)	−0.16439 (5)	0.15296 (6)	0.02110 (19)	
O5	0.01416 (8)	0.16353 (6)	0.42853 (7)	0.0296 (2)	
O6	0.16194 (8)	0.26344 (6)	0.55086 (7)	0.0251 (2)	
C1	0.13702 (11)	0.08557 (8)	0.28038 (9)	0.0170 (2)	
C2	0.03246 (11)	0.19213 (8)	0.12974 (9)	0.0184 (2)	
C3	−0.04554 (11)	0.10083 (8)	0.08528 (9)	0.0180 (2)	
C4	−0.02494 (10)	0.01226 (8)	0.14162 (9)	0.0168 (2)	
C5	0.21999 (12)	0.26756 (8)	0.26136 (10)	0.0272 (3)	
H5A	0.2792	0.2719	0.2198	0.041*	
H5B	0.2747	0.2586	0.3343	0.041*	
H5C	0.1671	0.3313	0.2531	0.041*	
C6	−0.10441 (11)	−0.08065 (8)	0.09481 (9)	0.0188 (2)	
C7	−0.15552 (12)	−0.25469 (9)	0.10665 (10)	0.0263 (3)	
H7A	−0.2505	−0.2422	0.0978	0.039*	
H7B	−0.1230	−0.3147	0.1517	0.039*	
H7C	−0.1465	−0.2681	0.0391	0.039*	
C8	0.23999 (11)	0.07215 (8)	0.38845 (9)	0.0191 (2)	
C9	0.38107 (12)	0.06442 (9)	0.37925 (11)	0.0272 (3)	
H9A	0.3937	0.1212	0.3362	0.041*	
H9B	0.3899	−0.0018	0.3475	0.041*	
H9C	0.4495	0.0689	0.4482	0.041*	
C10	0.21098 (13)	−0.02658 (8)	0.43885 (10)	0.0257 (3)	
H10A	0.2773	−0.0336	0.5084	0.038*	
H10B	0.2169	−0.0866	0.3971	0.038*	
H10C	0.1202	−0.0227	0.4432	0.038*	
C11	0.12831 (11)	0.19193 (8)	0.47419 (9)	0.0212 (3)	
C12	0.05270 (13)	0.30190 (10)	0.58204 (12)	0.0345 (3)	
H12A	0.0272	0.2496	0.6247	0.041*	
H12B	−0.0271	0.3166	0.5204	0.041*	
C13	0.09959 (12)	0.39926 (9)	0.64339 (10)	0.0244 (3)	
C14	0.01477 (13)	0.48439 (10)	0.62565 (11)	0.0355 (3)	
H14	−0.0716	0.4813	0.5736	0.043*	
C15	0.05509 (15)	0.57377 (10)	0.68321 (12)	0.0389 (4)	
H15	−0.0034	0.6319	0.6701	0.047*	
C16	0.17894 (14)	0.57887 (9)	0.75897 (11)	0.0332 (3)	
H16	0.2059	0.6402	0.7987	0.040*	
C17	0.26443 (12)	0.49493 (9)	0.77748 (10)	0.0269 (3)	
H17	0.3503	0.4983	0.8301	0.032*	
C18	0.22509 (11)	0.40592 (9)	0.71942 (10)	0.0225 (3)	
H18	0.2849	0.3486	0.7318	0.027*	
H3	0.3174 (15)	0.1854 (10)	0.4951 (12)	0.034 (4)*	

### Atomic displacement parameters (Å^2^)

**Table:**

	*U*^11^	*U*^22^	*U*^33^	*U*^12^	*U*^13^	*U*^23^
N1	0.0181 (4)	0.0182 (5)	0.0144 (5)	0.0006 (4)	0.0032 (4)	−0.0008 (4)
N2	0.0202 (5)	0.0163 (4)	0.0165 (5)	−0.0014 (4)	0.0036 (4)	0.0010 (4)
N3	0.0203 (5)	0.0181 (5)	0.0179 (6)	−0.0007 (4)	0.0015 (4)	−0.0041 (4)
O1	0.0277 (4)	0.0203 (4)	0.0230 (5)	0.0020 (3)	0.0052 (4)	0.0066 (4)
O2	0.0259 (4)	0.0229 (4)	0.0154 (5)	0.0030 (4)	0.0000 (4)	0.0016 (4)
O3	0.0215 (4)	0.0273 (4)	0.0199 (5)	−0.0012 (3)	−0.0006 (4)	−0.0012 (4)
O4	0.0229 (4)	0.0179 (4)	0.0205 (5)	−0.0035 (3)	0.0048 (4)	−0.0013 (3)
O5	0.0224 (4)	0.0287 (5)	0.0340 (6)	−0.0044 (3)	0.0046 (4)	−0.0112 (4)
O6	0.0226 (4)	0.0261 (4)	0.0266 (5)	−0.0024 (3)	0.0082 (4)	−0.0108 (4)
C1	0.0183 (5)	0.0157 (5)	0.0162 (6)	0.0022 (4)	0.0048 (5)	0.0004 (5)
C2	0.0191 (5)	0.0194 (5)	0.0165 (6)	0.0033 (4)	0.0058 (5)	0.0018 (5)
C3	0.0170 (5)	0.0217 (6)	0.0142 (6)	0.0044 (4)	0.0039 (5)	0.0003 (5)
C4	0.0159 (5)	0.0182 (5)	0.0154 (6)	0.0019 (4)	0.0040 (5)	−0.0004 (5)
C5	0.0293 (6)	0.0217 (6)	0.0260 (7)	−0.0075 (5)	0.0033 (6)	0.0011 (5)
C6	0.0174 (5)	0.0214 (6)	0.0175 (6)	0.0020 (4)	0.0056 (5)	−0.0007 (5)
C7	0.0293 (6)	0.0217 (6)	0.0270 (8)	−0.0084 (5)	0.0083 (6)	−0.0034 (5)
C8	0.0218 (5)	0.0157 (5)	0.0161 (6)	0.0017 (4)	0.0016 (5)	−0.0016 (5)
C9	0.0219 (6)	0.0276 (6)	0.0264 (7)	0.0045 (5)	0.0008 (6)	−0.0051 (6)
C10	0.0346 (6)	0.0174 (6)	0.0182 (7)	0.0016 (5)	0.0000 (6)	0.0008 (5)
C11	0.0248 (6)	0.0156 (5)	0.0209 (7)	−0.0010 (4)	0.0047 (5)	−0.0004 (5)
C12	0.0265 (6)	0.0389 (7)	0.0416 (9)	−0.0076 (6)	0.0162 (7)	−0.0178 (7)
C13	0.0228 (5)	0.0272 (6)	0.0248 (7)	−0.0021 (5)	0.0102 (5)	−0.0060 (5)
C14	0.0261 (6)	0.0450 (8)	0.0305 (8)	0.0079 (6)	0.0031 (6)	−0.0096 (7)
C15	0.0456 (8)	0.0335 (7)	0.0368 (9)	0.0159 (6)	0.0129 (7)	−0.0038 (6)
C16	0.0455 (8)	0.0265 (6)	0.0318 (8)	−0.0058 (6)	0.0185 (7)	−0.0094 (6)
C17	0.0254 (6)	0.0351 (7)	0.0214 (7)	−0.0069 (5)	0.0095 (6)	−0.0034 (5)
C18	0.0231 (6)	0.0236 (6)	0.0229 (7)	0.0012 (5)	0.0106 (5)	0.0020 (5)

### Geometric parameters (Å, º)

**Table:**

N1—C1	1.2923 (13)	C7—H7B	0.9800
N1—C4	1.3667 (15)	C7—H7C	0.9800
N2—C2	1.3833 (15)	C8—C10	1.5317 (15)
N2—C1	1.3943 (13)	C8—C9	1.5376 (15)
N2—C5	1.4797 (14)	C9—H9A	0.9800
N3—C11	1.3473 (14)	C9—H9B	0.9800
N3—C8	1.4694 (14)	C9—H9C	0.9800
N3—H3	0.875 (15)	C10—H10A	0.9800
O1—C2	1.2320 (13)	C10—H10B	0.9800
O2—C3	1.3390 (14)	C10—H10C	0.9800
O2—H2	0.876 (13)	C12—C13	1.5028 (17)
O3—C6	1.2239 (15)	C12—H12A	0.9900
O4—C6	1.3187 (13)	C12—H12B	0.9900
O4—C7	1.4507 (13)	C13—C18	1.3843 (18)
O5—C11	1.2109 (14)	C13—C14	1.3865 (17)
O6—C11	1.3562 (14)	C14—C15	1.3838 (19)
O6—C12	1.4457 (14)	C14—H14	0.9500
C1—C8	1.5255 (17)	C15—C16	1.369 (2)
C2—C3	1.4503 (16)	C15—H15	0.9500
C3—C4	1.3581 (15)	C16—C17	1.3781 (17)
C4—C6	1.4813 (15)	C16—H16	0.9500
C5—H5A	0.9800	C17—C18	1.3819 (16)
C5—H5B	0.9800	C17—H17	0.9500
C5—H5C	0.9800	C18—H18	0.9500
C7—H7A	0.9800		
			
C1—N1—C4	119.05 (10)	C1—C8—C9	108.47 (10)
C2—N2—C1	121.31 (9)	C10—C8—C9	109.24 (9)
C2—N2—C5	113.23 (9)	C8—C9—H9A	109.5
C1—N2—C5	125.38 (10)	C8—C9—H9B	109.5
C11—N3—C8	122.65 (10)	H9A—C9—H9B	109.5
C11—N3—H3	117.7 (9)	C8—C9—H9C	109.5
C8—N3—H3	118.5 (9)	H9A—C9—H9C	109.5
C3—O2—H2	102.2 (10)	H9B—C9—H9C	109.5
C6—O4—C7	114.62 (10)	C8—C10—H10A	109.5
C11—O6—C12	116.08 (9)	C8—C10—H10B	109.5
N1—C1—N2	122.28 (11)	H10A—C10—H10B	109.5
N1—C1—C8	116.74 (9)	C8—C10—H10C	109.5
N2—C1—C8	120.89 (9)	H10A—C10—H10C	109.5
O1—C2—N2	121.61 (10)	H10B—C10—H10C	109.5
O1—C2—C3	123.23 (11)	O5—C11—N3	126.06 (11)
N2—C2—C3	115.13 (10)	O5—C11—O6	124.26 (10)
O2—C3—C4	126.26 (10)	N3—C11—O6	109.67 (10)
O2—C3—C2	114.58 (10)	O6—C12—C13	108.10 (9)
C4—C3—C2	119.14 (11)	O6—C12—H12A	110.1
C3—C4—N1	122.78 (10)	C13—C12—H12A	110.1
C3—C4—C6	118.53 (11)	O6—C12—H12B	110.1
N1—C4—C6	118.69 (10)	C13—C12—H12B	110.1
N2—C5—H5A	109.5	H12A—C12—H12B	108.4
N2—C5—H5B	109.5	C18—C13—C14	118.59 (11)
H5A—C5—H5B	109.5	C18—C13—C12	121.68 (11)
N2—C5—H5C	109.5	C14—C13—C12	119.71 (12)
H5A—C5—H5C	109.5	C15—C14—C13	120.45 (13)
H5B—C5—H5C	109.5	C15—C14—H14	119.8
O3—C6—O4	123.35 (10)	C13—C14—H14	119.8
O3—C6—C4	121.54 (10)	C16—C15—C14	120.33 (12)
O4—C6—C4	115.10 (10)	C16—C15—H15	119.8
O4—C7—H7A	109.5	C14—C15—H15	119.8
O4—C7—H7B	109.5	C15—C16—C17	119.91 (12)
H7A—C7—H7B	109.5	C15—C16—H16	120.0
O4—C7—H7C	109.5	C17—C16—H16	120.0
H7A—C7—H7C	109.5	C16—C17—C18	119.93 (13)
H7B—C7—H7C	109.5	C16—C17—H17	120.0
N3—C8—C1	111.98 (9)	C18—C17—H17	120.0
N3—C8—C10	108.59 (9)	C17—C18—C13	120.79 (11)
C1—C8—C10	110.51 (9)	C17—C18—H18	119.6
N3—C8—C9	107.98 (9)	C13—C18—H18	119.6
			
C4—N1—C1—N2	−0.69 (14)	N1—C4—C6—O4	−2.56 (13)
C4—N1—C1—C8	−177.17 (9)	C11—N3—C8—C1	56.33 (14)
C2—N2—C1—N1	5.48 (15)	C11—N3—C8—C10	−65.98 (14)
C5—N2—C1—N1	−170.84 (10)	C11—N3—C8—C9	175.69 (11)
C2—N2—C1—C8	−178.18 (9)	N1—C1—C8—N3	−136.08 (10)
C5—N2—C1—C8	5.49 (15)	N2—C1—C8—N3	47.39 (13)
C1—N2—C2—O1	175.31 (9)	N1—C1—C8—C10	−14.88 (13)
C5—N2—C2—O1	−7.95 (14)	N2—C1—C8—C10	168.59 (9)
C1—N2—C2—C3	−6.35 (14)	N1—C1—C8—C9	104.85 (10)
C5—N2—C2—C3	170.39 (9)	N2—C1—C8—C9	−71.68 (12)
O1—C2—C3—O2	2.85 (15)	C8—N3—C11—O5	−11.11 (19)
N2—C2—C3—O2	−175.46 (8)	C8—N3—C11—O6	170.45 (10)
O1—C2—C3—C4	−178.65 (10)	C12—O6—C11—O5	3.79 (17)
N2—C2—C3—C4	3.04 (14)	C12—O6—C11—N3	−177.74 (10)
O2—C3—C4—N1	179.76 (9)	C11—O6—C12—C13	−164.02 (10)
C2—C3—C4—N1	1.45 (15)	O6—C12—C13—C18	−45.05 (16)
O2—C3—C4—C6	−1.11 (16)	O6—C12—C13—C14	136.37 (12)
C2—C3—C4—C6	−179.41 (9)	C18—C13—C14—C15	−0.22 (19)
C1—N1—C4—C3	−2.74 (15)	C12—C13—C14—C15	178.41 (12)
C1—N1—C4—C6	178.12 (9)	C13—C14—C15—C16	−0.5 (2)
C7—O4—C6—O3	−0.77 (14)	C14—C15—C16—C17	0.6 (2)
C7—O4—C6—C4	179.58 (8)	C15—C16—C17—C18	0.13 (19)
C3—C4—C6—O3	−1.39 (15)	C16—C17—C18—C13	−0.87 (18)
N1—C4—C6—O3	177.78 (9)	C14—C13—C18—C17	0.91 (17)
C3—C4—C6—O4	178.27 (9)	C12—C13—C18—C17	−177.69 (10)

### Hydrogen-bond geometry (Å, º)

**Table:**

*D*—H···*A*	*D*—H	H···*A*	*D*···*A*	*D*—H···*A*
O2—H2···O3	0.876 (13)	1.782 (14)	2.5879 (12)	151.9 (14)
N3—H3···O1^i^	0.875 (15)	2.118 (15)	2.9854 (16)	170.9 (12)

Symmetry code: (i) *x*+1/2, −*y*+1/2, *z*+1/2.
